# Associations between housing tenure and unscheduled hospital use in people of working age with multiple physical and mental long-term conditions: a retrospective cohort study using linked data from London

**DOI:** 10.1186/s12913-026-15071-3

**Published:** 2026-07-20

**Authors:** Preveina Mahadevan, Helen A. Blake, Jenny Shand, Manuel Gomes, Jessica Sheringham

**Affiliations:** 1 Research Department of Primary Care and Population Health, University College London, London, WC1E 6BT UK; 2 Clinical, Educational and Health Psychology, University College London, London, WC1E 6BT UK

**Keywords:** Healthcare inequalities, Hospital admissions, Multiple long-term conditions, Housing tenure

## Abstract

**Background:**

Individuals with physical and mental long-term conditions typically experience high levels of hospital use. Housing tenure – the arrangements under which individuals occupy their home – has been shown to be associated with the co-occurrence of physical and mental long-term conditions. However, the association between housing tenure and unplanned hospital use is not established. This study investigates the longitudinal relationship between housing tenure and unplanned hospital admissions among individuals of working age with co-occurring physical and mental long-term conditions.

**Methods:**

A longitudinal retrospective cohort study was undertaken, utilising the Care City Cohort, a dataset comprising healthcare, social care and wider determinants variables for residents in North East London. Two-part regression models with multiple imputation were used to estimate the odds of experiencing an unscheduled hospital admission and the associated length of stay among individuals with co-occurring physical and mental long-term conditions, by housing tenure. The models were adjusted for socio-demographic characteristics, health-related behaviours, and the number of physical and mental long-term conditions.

**Results:**

Analysis was conducted on the records of 45,399 individuals diagnosed with physical and mental long-term conditions before April 2016. The most common tenure was owner-occupied was 48%, followed by social renting (27.1%) and privately rented housing (24.9%). After adjusting for sociodemographic variables and health vulnerabilities, the odds of a non-elective admission were higher for social housing residents (OR 1.40; 95%CI: 1.21–1.61) than owner-occupied. There was no evidence of different lengths of stay between tenures.

**Conclusions:**

This study highlights housing tenure as an important determinant of unscheduled hospital admissions amongst working age adults with a combination of physical and mental long-term conditions. Housing tenure status may be a useful indicator for local services to target secondary prevention strategies to reduce unscheduled hospital use.

**Supplementary Information:**

The online version contains supplementary material available at https://doi.org/10.1186/s12913-026-15071-3.

## Background

The public health burden of multiple long-term conditions is well recognised and growing. Individuals with multiple long-term conditions experience unplanned hospitalisations nearly five times more frequently and have longer lengths of hospital stays than people with one long-term condition [1–4]. The co-occurrence of a physical and a mental long-term condition (PMLTCs) has increased globally [5–8]. People with this combination of conditions have particularly high use of unscheduled hospital care. In addition to the burden on patients, these outcomes add considerable costs to the health and care system [2, 9].

Household-level markers of socioeconomic circumstances, often overlooked in multiple long-term condition studies, are associated with prevalence [10]. In particular, housing tenure – a household’s relationship with their home – can be highly correlated with prevalence [10]. In England, owner-occupation is the most common tenure (65% of all households), followed by private rented (19%) and socially rented (16%). London has a distinct tenure profile with almost double the proportion of private renters to the rest of the country (32% vs. 19%) [11].

Housing tenure is influenced by sociodemographic and economic circumstances. It is also highly correlated with housing quality [12], which can affect health [10, 13–18]. Social housing renters have lower well-being scores than other tenure types [19]. However, private renters may have the least agency to affect their living conditions. For example, compared with owner-occupiers or social renters, private renters have the highest proportion of non-decent dwellings, dampness and lower average energy efficiency rating [11].

Few studies, however, have examined associations between housing tenure and unscheduled hospital use amongst individuals with PMLTCs [3, 20, 21]. Although some research has focused on PMLTCs in older populations, there are increasing calls to focus on younger populations in PMLTCs research, where distinct issues arise for working-age populations in accessing healthcare compared with adults above the statutory retirement age [16].

The objective of this paper is to examine the association between household tenure and unplanned hospital use amongst people of working age with PMLTCs. Our first hypothesis was that individuals with PMLTCs living in social housing are more likely to be admitted to hospital as unscheduled admissions than individuals who own their home. We hypothesised also that length of stay would be longer for private renters (who would be less able to alter their home environment), than owner occupiers.

## Methods

### Design

We conducted a retrospective cohort study using a linked dataset of healthcare and wider determinants records from residents living in the borough of Barking & Dagenham in North East London.

### Data

The Care City Cohort dataset comprises linked administrative healthcare and wider determinants data collected by London Borough of Barking and Dagenham (LBBD), health providers, and commissioners [22]. Details on how dataset was created and its composition can be found in Shand et al. 2020 [22]. The dataset comprised patients living in the borough of Barking and Dagenham, a socioeconomically disadvantaged borough of North East London, with approximately 210,000 residents and a younger and more ethnically diverse population than England [23]. The dataset was ordered chronologically by patient identifier and individuals with duplicate records were removed by taking the first record of each duplicate.

Exposure, covariates, and outcomes data were collected from 2017 to 2019, as these years were the latest available data from the Care City Cohort database and better reflective of the cohort in real time.

### Cohort

The study cohort was individuals aged between 15 and 64 years old [19], diagnosed with PMLTCs before 1st April 2016. A flowchart for how the cohort was constructed via data linkages, is given in Supplementary Fig. 1. The definition of PMLTCs was adapted from the Academy of Medical Sciences definition [24]. Individuals were included in the cohort if they had a diagnosis recorded in primary care data for both a physical conditions and common mental health condition (for a full list of eligible conditions, please see Supplementary Table 1, file 2). Physical long-term conditions were quantified using an unweighted count of physical health conditions (0, 1, 2, 3, or ≥ 4). Mental health conditions consisted of common mental illnesses defined by National Institute for Health and Care Excellence guidance [25]. The presence of any common mental health condition was included as a separate binary variable (≥ 1 or 0) because some individuals were diagnosed with more than one condition. We opted to group depression and anxiety diagnoses as one binary variable in part for simplicity and in part because of evidence that patients can experience significant flux between depression and anxiety diagnoses [26].

### Exposure

The exposure was household tenure, grouped into three categories: owner-occupied, privately rented, and social housing. Data on tenure was extracted from LBBD property records from 2017. The reference group was owner-occupied housing tenure. Individuals were classified as owner-occupied if living in a house that was owned outright or with a mortgage. Individuals in social housing properties were identified from the council’s social housing data system. Individuals in privately-rented properties were identified from the council’s private rental licensing system. Residents renting privately but in affordable, subsidised rental properties were grouped with social renters. Ten individuals classified as having unknown tenure were excluded from the cohort.

### Outcomes

Unscheduled hospital use was defined as: the number of non-elective admissions between 2017 and 2019 and the length of stay of non-elective admissions. In England’s National Health Service (NHS), a non-elective admission is defined as a hospital admission that is not planned in advance and requires an overnight stay of at least one day [27]. Patients were admitted through emergency departments or via direct urgent referrals from healthcare professionals. Data on non-elective admissions originated from the NHS secondary uses database and were linked to the study cohort using NHS number. The length of stay was measured by the difference in days between the patient’s hospital admission and discharge.

### Covariates

Based on the previously published related literature [12, 18, 31–33] and data availability, variables that may affect tenure decisions and hospitalisation were included as covariates adjusted models:

- Individual and area-level social characteristics: gender, age, ethnicity, Index of Multiple of Deprivation 2019 (IMD).
- Health-related behaviours: Body Mass Index (BMI), smoking status, recorded alcohol intake problems.
- Indicators of pre-existing illness: number of physical-mental long-term conditions.
- Household occupancy: number of inhabitants in a household, categorised as 1 (lone occupancy), 2–4, 5–9 or 10–12 individuals.

Continuous covariates were categorised based on how the values were distributed. IMD deciles six to ten (the least deprived) were merged due to small numbers.

For a full description of all the variables, please see Supplementary Table 2, file 2.

### Analysis

All analysis was conducted using R [28]. A descriptive analysis was conducted to summarise the characteristics of the cohort and on the number of individuals who had at least one non-elective admission in a particular year, as a percentage of the total number of individuals with PMLTCs for each covariate. The analysis was conducted on individuals with complete data on physical and mental health conditions.

A two-part model [29] using the twopartm package in R software was used to investigate the association between housing tenure and non-elective admissions and length of stay (LoS). This approach is recommended for the analysis of healthcare utilisation because a high proportion of individuals who do not experience an admission (i.e. have zero utilisation) [29]. Two-part models recognise that individuals with zero healthcare utilisation (non-users) are systematically different from health service users. The approach involves first estimating the probability of having a non-zero outcome (in this case a non-elective hospital admission) via probit or logit and subsequently modelling the outcome of interest (hospital length-of-stay in this study), conditional on having a non-zero outcome via a generalised linear model. A substantial proportion of people had zero hospital length of stay and, by definition, zero hospital admissions.

To assess the association between housing tenure and unscheduled hospital stay, two models were fitted. For transparency, an unadjusted model with no covariates was first fitted. Second, a model was fitted adjusted for sociodemographic variables associated with both household tenure, PMLTCs and healthcare utilisation that were available in the dataset. These covariates were age, gender, ethnicity, IMD 2019, calendar year, BMI, smoking status, recorded alcohol intake, the number of diagnosed physical and mental long-term conditions and household occupancy. Multiple imputation was conducted on variables with missing data, including BMI, ethnicity, and smoking status.

The number of imputations was based on the percentage of incomplete records [30]. In this dataset, around 30% of the records had at least one missing value hence 30 imputations were conducted. Other variables that may affect the relationship between housing tenure and hospital visits were used as auxiliary variables to estimate imputations. Rubin’s rules were used to pool results across imputed datasets. Monte Carlo errors for all pooled coefficients were less than 10% of their corresponding standard errors (Supplementary Table 4, file 2) as recommended by White et al. 2011 [30]. This provides corroborative evidence that our findings would remain even if the MI process was repeated.

### Sensitivity Analysis

A complete case analysis was conducted to assess the robustness of the findings derived from the main analysis. The underlying assumption for this analysis was that any differences between individuals with missing and observed data were explained by those covariates included in the model.

### Involvement of lived experience perspectives

Early study plans were shared Care City Community Board, comprising Barking & Dagenham residents, patients, carers and staff from the voluntary sector, care and health agencies, for review and to obtain approval to access the data. Interim findings were shared with the Community Board to inform the interpretation of results.

## Results

### Descriptive analysis

Analysis was conducted on the records of 45,399 distinct individuals and 29,767 households with diagnosed PMLTCs before April 2016. The most common tenure in Care City was owner-occupied (48.5%), followed by social housing (28.7%) and private rental (22.9%). The demographic and health characteristics of individuals varied by tenure. There was highest prevalence of three or over long-term conditions in social housing (19.1%) and lowest in private rentals (11.3%). The highest proportion of residents living on their own was found in social housing (12.3%) compared with 7.5% in owner occupied housing.

Out of the total cohort, 2263 (5.0%) individuals had at least one non-elective admission by 2019. (Table 1), with social renters having the highest proportion of patients that had non-elective admissions (6.5%) and the lowest proportion in owner-occupied households (4.2%). Average lengths of stay were 4 days overall with longer durations for privately-renting residents (4.3 days) and social housing residents (4.1 days), and shorter lengths of stay for owner-occupying residents (3.7 days).

**Table 1:** Summary characteristics of patients from the cohort in 2019, by housing tenure category (*N* = 45399)

Characteristic	Total *N* (%)	Type of Housing tenure *N* (%)		
		Owner-occupied (*N* = 22006)	Privately rented (*N* = 10380)	Social Housing (*N* = 13013)
***Individual-level variables***				
**Sex**				
Female	24,472 (53.90)	11,445(52.01)	5652(54.45)	7375(56.67)
Male	20,927 (46.10)	10,561(47.99)	4728(45.55)	5638(43.33)
**Age**				
15–24	2793 (6.15)	1181(5.37)	706(6.80)	906(6.96)
25–34	5160 (11.37)	2338(10.62)	1057(10.18)	1765(13.56)
35–44	10,791 (23.77)	4600(20.90)	3119(30.05)	3072(23.61)
45–54	13,017 (28.67)	6381(29.00)	3222(31.04)	3414(26.24)
55–64	13,638 (30.04)	7506(34.11)	2276(21.93)	3856(29.63)
**Ethnicity**				
White	16,341 (35.99)	7132(32.41)	3393(32.69)	5816(44.69)
Asian	7185 (15.83)	4597(20.89)	1947(18.76)	641(4.93)
Black	7176 (15.81)	3038(13.81)	2227(21.45)	1911(14.69)
Mixed	1065 (2.35)	419(1.90)	305(2.94)	341(2.62)
Other	805 (1.77)	419(1.90)	266(2.56)	120(0.92)
Unknown	12,827 (28.25)	6401(29.09)	2242(21.60)	4184(32.15)
**BMI**				
Underweight	1465 (3.23)	625(2.84)	356(3.43)	484(3.72)
Healthy weight	10,526 (23.19)	5249(23.85)	2509(24.17)	2768(21.27)
Overweight	12,981 (28.59)	6624(30.10)	2998(28.88)	3359(25.81)
Obese	11,422 (25.16)	5450(24.77)	2489(23.98)	3483(26.77)
Morbidly obese	8989 (19.80)	4051(18.41)	2027(19.53)	2911(22.37)
Unknown	16 (0.04)	7(0.03)	1(0.01)	8(0.06)
**Smoking Status**				
Non-smoker	27,936 (61.53)	14,908(67.75)	6433(61.97)	6595(50.68)
Ex-smoker	6930 (15.26)	3303(15.01)	1390(13.39)	2237(17.19)
Smoker	9277 (20.43)	3267(14.85)	2225(21.44)	3785(29.09)
Unknown	1256 (2.77)	528(2.40)	332(3.20)	396(3.04)
**Alcohol Consumption**				
Yes	573(1.26)	158(0.72)	119(1.15)	296(2.27)
No	44,826(98.74)	21,848(99.28)	10,261(98.85)	12,717(97.73)
**No. physical & mental long term conditions**				
1	14,239 (31.36)	6661(30.27)	3679(35.44)	3899(29.96)
2–3	24,809 (54.65)	12,394(56.32)	5582(53.78)	6833(52.51)
4–5	5679 (12.51)	2703(12.28)	1010(9.73)	1966(15.11)
> 5	672 (1.48)	248(1.13)	109(1.05)	315(2.42)
**Non-elective admissions**				
0	43,136(95.02)	21,085(95.81)	9891(95.29)	12,160(93.45)
1+	2263(4.98)	921(4.19)	489(4.71)	853(6.55)
**Average length of stay** **(days**,** SD)**	3.97(6.87)	3.66(6.01)	4.30(9.34)	4.09(6.05)
***Household-level variables***				
**Household Occupancy**				
1	4261 (9.39)	1655(7.52)	1011(9.74)	1595(12.26)
2–4	26,577 (58.54)	13,314(60.50)	5534(53.31)	7729(59.39)
5–9	13,984 (30.80)	6749(30.67)	3602(34.70)	3633(27.92)
10–12	577 (1.27)	288(1.31)	233(2.24)	56(0.43)
**Index of Multiple of Deprivation (IMD)**				
1	2139 (4.71)	507(2.30)	327(3.15)	1305(10.03)
2	24,592 (54.17)	10,606(48.20)	5319(51.24)	8667(66.60)
3	13,057 (28.76)	6718(30.53)	3651(35.17)	2688(20.66)
4	2598 (5.72)	1822(8.28)	600(5.78)	176(1.35)
5	2345 (5.17)	1804(8.20)	370(3.56)	171(1.31)
6–10	668 (1.47)	549(2.49)	113(1.09)	6(0.05)

*<< Summary characteristics of patients from the cohort in 2019*,* by housing tenure category >>*.

### Household tenure and non-elective hospital admissions

After adjusting for sociodemographic variables and health vulnerabilities and imputing the variables with missing data, there was strong evidence that the odds of a non-elective admission remained higher for social housing residents (AOR 1.40; 95% CI:1.21–1.61) than owner-occupiers. There was no evidence for different lengths of stay by housing tenure (Table 2).

**Table 2:** Risk of non-elective admission and associations with hospital stay duration by housing tenure (using data from multiple imputation): two part model

Housing Tenure	Unadjusted		Adjusted^a^	
	PART 1-Logit OR (95 CI%)	PART 2 -GLM Coefficient (95 CI%)	PART 1-Logit OR (95 CI%)	PART 2 -GLM Coefficient (95 CI%)
Owner- occupied (ref)				
Social Housing	1.73(1.52–1.97)***	0.43(-0.45-1.31)	1.40(1.21–1.61)***	0.26(-0.73-1.26)
Privately rented	1.14(0.98–1.33).	0.64(-0.41-1.69)	1.10(0.94–1.29)	0.58(-0.51-1.67)

^a^Model adjusted for: Age, Gender, Ethnicity, Index of Multiple Deprivation (IMD), Year, Body Mass Index (BMI), smoking status, recorded alcohol intake, Number of physical-mental long-term conditions, Household occupancy

GLM coefficients refer to the average length of stay

***p-value 0.00 **p-value 0.001 *p-value 0.01. p-value 0.1

### Sensitivity analysis

Complete case analysis showed similar results to the main results, with similar effect estimates (Supplementary Table 3, file 2).

## Discussion

### Summary of main findings

In this retrospective cohort study of working age adults with physical and mental health conditions in East London, residents of social rental sectors had higher odds of unscheduled hospital admissions than individuals who owned their homes. Associations remained after adjusting for health vulnerabilities and socio-demographic characteristics. There was no evidence for longer lengths of stay amongst social housing residents than other tenures after adjusting for other variables.

### Strengths and limitations

The main strength of this study lies in the comprehensiveness of the Care City Cohort, which enables data linkage to the local authority dataset and allows for the exploration of tenure data at the household level. The database was granular enough to allow us to examine deprivation in urban settings using social housing data and investigate its impact on healthcare utilisation and the associations between social determinants with healthcare use and outcomes. Additionally, the study incorporates insights from lived experiences, enhancing its applicability to practical, day-to-day settings, which helped to contextualise experiences of living with multiple conditions and possible influences of household tenure on healthcare use.

The study was conducted in one borough of East London, with a distinct ethnicity and socioeconomic profile. Therefore, these findings may not be generalisable to other areas of the UK or other countries, e.g. where there are different drivers of hospital use or where eligibility for social housing is different.

Other limitations include the potential for residual confounding because the dataset did not include potentially relevant information such as resident occupation and housing conditions. We acknowledge the potential for clustering within households, and taking this into account, would increase the precision of the results [31]. It was outside the scope of this study to examine this, but it is a potentially relevant consideration for future work. It was also out of scope to examine cause of hospitalisation, which might have indicated if exacerbations of current physical or mental health conditions were the cause of admissions.

Multiple imputation was conducted to account for missing data. Ethnicity had the highest level of missing data (28%). In the UK, housing tenure is strongly associated with ethnicity, with higher proportions of minority ethnic groups living in social housing than white English groups, so more complete ethnicity data would be important to examine potential effect modification of the relationship between tenure and healthcare use [11]. 

### Possible explanations for our findings

Many studies aim to explain higher unscheduled hospital admissions by examining associations with socioeconomic disadvantage and deprivation [32–34]. Housing tenure may be acting as a proxy for socioeconomic disadvantage, which is consistently associated with higher unscheduled healthcare use [35–37]. While it is not straightforward to compare findings from different studies, the strength of association between unscheduled admissions and housing tenure in this study was larger than area-level deprivation indices (e.g. Bankart et al. reported an increased rate of admissions of 1.02; 95% CI 1.01–1.02) by Index of Multiple Deprivation) [38]. The strength of association found in our study (AOR 1.40; 95% CI 1.21–1.61) was also similar to the risk of frequent hospital admissions reported by Low et al. for individuals living in public rental housing versus other tenures (AOR 1.30; 95% CI 1.13–1.50) [36].

Murphy and Grundy (2024) conceptualise housing tenure as a proxy for socioeconomic position, and – in common with our analysis – find it is more strongly associated with poor health than other socioeconomic markers, particularly in the UK context [39]. We explore some of the potential explanations proposed by Murphy and Grundy to explore why tenure was associated with emergency hospital admissions.

Firstly, there is the possibility of a selection effect. Poor health is one reason for eligibility for social housing, so people in this tenure are already more likely to be in poor health. Whilst we included variables for additional comorbidities and health behaviours, we did not have data on severity of conditions. Relatedly, they propose that health behaviours by tenure may be relevant. Other studies have reported stark inequalities in smoking and quitting rates by tenure, with residents in social housing having over two times the odds of smoking as owner occupiers [40]. However, in common with the analysis of multiple long term condition prevalence [10], we found associations remained after controlling for health behaviours.

Secondly, it is possible that poorer housing conditions, more often found in privately and socially rented than owner-occupied tenures, increase the risk of ill health, necessitating an emergency admission. Although social housing is more likely to meet decent home standards than privately rented homes [41], individuals living in social housing are still more likely to face adverse housing conditions than owner-occupiers [42]. There is evidence that poor living conditions such as overcrowding increase the risk of dampness and mould and can cause respiratory problems [43–45]. Previous research indicates that poor housing conditions like overcrowding can exacerbate pre-existing conditions [46].

A further explanation proposed by lived experience resident contributors was that working individuals with multiple conditions could be motivated to seek unscheduled healthcare differently than those not in employment to maintain their working lives. Working-age adults renting privately are more likely to be in employment than social housing tenants [42]. They also face higher rents and higher risk of eviction than social housing tenants. They may, therefore, avoid using emergency care whenever possible and when in hospital, feel an urgency to leave as soon as possible to return to work to prevent loss of income. This may explain why we did not find longer lengths of stay in private renters after adjusting for other factors, even though private renters have the least agency to adjust their home environment to accommodate changing needs following severe illness after a hospital stay [47, 48]. We did not have the data to explore these potential explanations but we recommend they are considered in future studies.

### Implications for research and practice

This study underlines the importance of household tenure in the UK as a determinant of healthcare use, amongst working age adults with long term conditions. Public services (including English integrated care systems) seeking to alleviate strain on healthcare services could target secondary prevention activities using household tenure information. Housing tenure information is widely available at a household level in many settings including the UK. Record linkage can be used to access this information reducing the needs for bespoke data collection and reducing the burden on health care workers. To inform such prevention activities, further studies are needed to estimate the cost implications of increased unscheduled healthcare utilisation and advance our understanding of the pathways by which housing tenure may influence healthcare utilisation amongst people with multiple long-term conditions.

## Supplementary Information

Below is the link to the electronic supplementary material.

Supplementary Material 1: Figure 1, file 1. Data flows and linkages

Supplementary Material 2: a. Supplementary Table 1. Definitions of mental and physical health conditions included in the cohortb. Supplementary Table 2. Variables included in the analysis. c. Supplementary Table 3. Sensitivity Analysis: Two-part model using a complete case analysis to assess non-elective hospital admission risk and stay duration. d. Supplementary Table 4. Results of Monte Carlo errors for all pooled coefficients in the Multiple Imputation

## Data Availability

Restrictions apply to the availability of these data. Data were obtained from Care City, following approval of the study protocol.

## Abbreviations

BMI: Body mass index
IMD: Index of multiple deprivation
LBBD: London Borough of Barking and Dagenham
OR: Odds ratio
PMLTC: Physical and mental long-term conditions (used in this paper for those with co-occurring mental and physical condition)
LoS: Length of stay
NIHR: National Institute for Health and Care Research
NHS: National Health Service

## References

[1] Payne RA, Abel GA, Guthrie B, Mercer SW. The effect of physical multimorbidity, mental health conditions and socioeconomic deprivation on unplanned admissions to hospital: a retrospective cohort study. CMAJ. 2013;185(5):E221–8. 10.1503/cmaj.121349 PMC3602270 23422444

[2] Soley-Bori M, Ashworth M, Bisquera A, Dodhia H, Lynch R, Wang Y, Fox-Rushby J. Impact of multimorbidity on healthcare costs and utilisation: a systematic review of the UK literature. Br J Gen Pract. 2021;71(702):e39–46. 10.3399/bjgp20X713897 PMC7716874 33257463

[3] Aubert CE, Schnipper JL, Fankhauser N, Marques-Vidal P, Stirnemann J, Auerbach AD, et al. Patterns of multimorbidity in medical inpatients: a multinational retrospective cohort study. Intern Emerg Med. 2020;15(7):1207–17. 10.1007/s11739-020-02306-2 32180102

[4] Rodrigues LP, França DG, Vissoci JRN, Caruzzo NM, Batista SR, de Oliveira C, et al. Associations of hospitalisation – admission, readmission and length to stay – with multimorbidity patterns by age and sex in adults and older adults: the ELSI-Brazil study. BMC Geriatr. 2023;23(1):504. 10.1186/s12877-023-04167-8 PMC10441711 37605111

[5] McLean G, Gunn J, Wyke S, Guthrie B, Watt GC, Blane DN, Mercer SW. The influence of socioeconomic deprivation on multimorbidity at different ages: a cross-sectional study. Br J Gen Pract. 2014;64(624):e440–7. 10.3399/bjgp14X680545 PMC4073730 24982497

[6] Chowdhury SR, Chandra Das D, Sunna TC, Beyene J, Hossain A. Global and regional prevalence of multimorbidity in the adult population in community settings: a systematic review and meta-analysis. eClinicalMedicine. 2023;57. 10.1016/j.eclinm.2023.101860 PMC9971315 36864977

[7] Wang Y-P, Nunes BP, Coêlho BM, Santana GL, do Nascimento CF, Viana MC, et al. Multilevel Analysis of the Patterns of Physical-Mental Multimorbidity in General Population of São Paulo Metropolitan Area, Brazil. Sci Rep. 2019;9(1):2390. 10.1038/s41598-019-39326-8 PMC6382818 30787376

[8] Taloyan M, Alinaghizadeh H, Wettermark B, Jan Hasselström JH, Bertilson BC. Physical-mental multimorbidity in a large primary health care population in Stockholm County, Sweden. Asian J Psychiatr. 2023;79:103354. 10.1016/j.ajp.2022.103354 36463815

[9] Cicek M, Hayhoe B, Otis M, Nicholls D, Majeed A, Greenfield G. Depression and unplanned secondary healthcare use in patients with multimorbidity: A systematic review. PLoS ONE. 2022;17(4):e0266605. 10.1371/journal.pone.0266605 PMC8989325 35390086

[10] Ingram E, Gomes M, Hogarth S, McDonald HI, Osborn D, Sheringham J. Household Tenure and Its Associations with Multiple Long-Term Conditions amongst Working-Age Adults in East London: A Cross-Sectional Analysis Using Linked Primary Care and Local Government Records. Int J Environ Res Public Health. 2022;19(7). 10.3390/ijerph19074155 PMC8998986 35409849

[11] Ministry of Housing Communities and Local Government. English Housing Survey 2023 to 2024: headline findings on demographics and household resilience. London. 2024. Accessed online at: https://doc.ukdataservice.ac.uk/doc/9443/mrdoc/pdf/9443_2023-24_ehs_headline_report_on_household_demographics_and_resilience.pdf

[12] Evans GW, Wells NM, Chan HY, Saltzman H. Housing quality and mental health. J Consult Clin Psychol. 2000;68(3):526–30. 10.1037//0022-006x.68.3.526 10883571

[13] Ingram E, Ledden S, Beardon S, Gomes M, Hogarth S, McDonald H, et al. Household and area-level social determinants of multimorbidity: a systematic review. J Epidemiol Community Health. 2021;75(3):232–41. 10.1136/jech-2020-214691 PMC7892392 33158940

[14] Wee LE, Low LL, Thumboo J, Chan A, Lee KH. Factors associated with emergency room visits and hospitalisation amongst low-income public rental flat dwellers in Singapore. BMC Public Health. 2019;19(1):713. 10.1186/s12889-019-7009-5 PMC6556005 31174499

[15] Dal Grande E, Chittleborough CR, Wu J, Shi Z, Goldney RD, Taylor AW. Effect of social mobility in family financial situation and housing tenure on mental health conditions among South Australian adults: results from a population health surveillance system, 2009 to 2011. BMC Public Health. 2015;15(1):675. 10.1186/s12889-015-2022-9 PMC4504347 26184770

[16] Al-Hanawi MK, Chirwa GC. Economic Analysis of Inequality in Preventive Health Check-Ups Uptake in Saudi Arabia. Front Public Health. 2021;9:745356. 10.3389/fpubh.2021.745356 PMC8484695 34604166

[17] Pollack CE, Griffin BA, Lynch J. Housing affordability and health among homeowners and renters. Am J Prev Med. 2010;39(6):515–21. 10.1016/j.amepre.2010.08.002 21084071

[18] Baker E, Bentley R, Mason K. The Mental Health Effects of Housing Tenure: Causal or Compositional? Urban Stud. 2013;50(2):426–42.

[19] OECD. Working-age population (15–64). Paris. Accessed online at: https://www.oecd.org/en/data/indicators/working-age-population.html

[20] Stahl-Toyota S, Nikendei C, Nagy E, Bönsel S, Rollmann I, Unger I, et al. Interaction of mental comorbidity and physical multimorbidity predicts length-of-stay in medical inpatients. PLoS ONE. 2023;18(6):e0287234. 10.1371/journal.pone.0287234 PMC10287009 37347745

[21] Rodrigues LP, de Oliveira Rezende AT, Delpino FM, Mendonça CR, Noll M, Nunes BP et al. Association between multimorbidity and hospitalization in older adults: systematic review and meta-analysis. Age Ageing. 2022;51(7). 10.1093/ageing/afac155 PMC9308991 35871422

[22] Shand J. Towards Integrated Care: Using Linked Data to Explore Health and Social Care Utilisation for Adult Residents of Barking and Dagenham in 2016/17. [PhD]. London: University College London (UCL); 2020.

[23] London Borough of Barking and Dagenham. Joint Strategic Needs Assessment. 2023.Accessed online at: B&D JSNA 2023-24.pdf.

[24] Academy of Medical Sciences. Multimorbidity: a priority for global health research. Academy of Medical Sciences. 2018. Accessed online at: https://acmedsci.ac.uk/file-download/82222577

[25] National Institute for Health and Care Excellence (NICE). Common mental health problems: identification and pathways to care. 2011. Accessed online at: https://www.nice.org.uk/guidance/cg123

[26] Ter Meulen WG, Draisma S, van Hemert AM, Schoevers RA, Kupka RW, Beekman ATF, Penninx BWJH. Depressive and anxiety disorders in concert-A synthesis of findings on comorbidity in the NESDA study. J Affect Disord. 2021;284:85–97. 10.1016/j.jad.2021.02.004 33588240

[27] NHS Data Model and Dictionary. 2005. Admitted patient total non-elective admissions. Accessed online at: https://archive.datadictionary.nhs.uk/DD%20Release%20March%202013/data_dictionary/data_field_notes/a/ad/admitted_patient_total_non-elective_admissions_de.asp@shownav=0.html

[28] R Core Team. R: A Language and Environment for Statistical Computing. Vienna, Austria: R Foundation for Statistical Computing; 2024. https://www.R-project.org/. v12.1 + 563 ed2024.

[29] Farewell VT, Long DL, Tom BDM, Yiu S, Su L. Two-Part and Related Regression Models for Longitudinal Data. Annu Rev Stat Appl. 2017;4:283–315. 10.1146/annurev-statistics-060116-054131 PMC5590716 28890906

[30] White IR, Royston P, Wood AM. Multiple imputation using chained equations: Issues and guidance for practice. Stat Med. 2011;30(4):377–99. 10.1002/sim.4067 21225900

[31] Stafford M, Deeny SR, Dreyer K, Shand J. Multiple long-term conditions within households and use of health and social care: a retrospective cohort study. BJGP Open. 2021;5(2). 10.3399/BJGPO.2020.0134 PMC8170597 33234513

[32] O’Cathain A, Knowles E, Maheswaran R, Pearson T, Turner J, Hirst E, et al. A system-wide approach to explaining variation in potentially avoidable emergency admissions: national ecological study. BMJ Qual Saf. 2014;23(1):47–55. 10.1136/bmjqs-2013-002003 PMC3888597 23904507

[33] McCormick B, Hill PS, Redding S. Comparative morbidities and the share of emergencies in hospital admissions in deprived areas: a method and evidence from English administrative data. BMJ Open. 2018;8(8):e022573. 10.1136/bmjopen-2018-022573 PMC6104760 30127052

[34] Warner M, Burn S, Stoye G, Aylin PP, Bottle A, Propper C. Socioeconomic deprivation and ethnicity inequalities in disruption to NHS hospital admissions during the COVID-19 pandemic: a national observational study. BMJ Qual Saf. 2022;31(8):590–8. 10.1136/bmjqs-2021-013942 PMC8627367 34824162

[35] Johnson L, Cornish R, Boyd A, Macleod J. Socio-demographic patterns in hospital admissions and accident and emergency attendances among young people using linkage to NHS Hospital Episode Statistics: results from the Avon Longitudinal Study of Parents and Children. BMC Health Serv Res. 2019;19(1):134. 10.1186/s12913-019-3922-7 PMC6390368 30808346

[36] Low LL, Tay WY, Ng MJ, Tan SY, Liu N, Lee KH. Frequent hospital admissions in Singapore: clinical risk factors and impact of socioeconomic status. Singap Med J. 2018;59(1):39–43. 10.11622/smedj.2016110 PMC5778257 27311740

[37] Low LL, Wah W, Ng MJ, Tan SY, Liu N, Lee KH. Housing as a Social Determinant of Health in Singapore and Its Association with Readmission Risk and Increased Utilization of Hospital Services. Front Public Health. 2016;4:109. 10.3389/fpubh.2016.00109 PMC4884736 27303662

[38] Bankart MJ, Baker R, Rashid A, Habiba M, Banerjee J, Hsu R, et al. Characteristics of general practices associated with emergency admission rates to hospital: a cross-sectional study. Emerg Med J. 2011;28(7):558–63. 10.1136/emj.2010.108548 21515879

[39] Murphy M, Grundy EMD. Housing tenure and disability in the UK: trends and projections 2004–2030. Front Public Health. 2024;Volume 11–2023. 10.3389/fpubh.2023.1248909 PMC10795505 38239788

[40] Jackson SE, Cheeseman H, Arnott D, Titmarsh R, Brown J. Smoking in social housing among adults in England, 2015–2020: a nationally representative survey. BMJ Open. 2022;12(7):e061013. 10.1136/bmjopen-2022-061013 PMC9330323 35882456

[41] Department for Levelling Up Housing and Communities. English Housing Survey 2021 to 2022: headline report. 2022. Accessed online at: https://www.gov.uk/government/statistics/english-housing-survey-2021-to-2022-headline-report/english-housing-survey-2021-to-2022-headline-report

[42] Department for Levelling Up Housing and Communities. English Housing Survey 2020 to 2021: headline report. 2021. Accessed online at: https://www.gov.uk/government/statistics/english-housing-survey-2020-to-2021-headline-report

[43] Holden KA, Lee AR, Hawcutt DB, Sinha IP. The impact of poor housing and indoor air quality on respiratory health in children. Breathe. 2023;19(2):230058. 10.1183/20734735.0058-2023 PMC10461733 37645022

[44] UK Government. Update on government’s work to improve the quality of social housing. Department for Levelling Up, Housing and Communities. 2023. Accessed online at: https://www.gov.uk/guidance/june-2023-update-on-governments-work-to-improve-the-quality-of-social-housing

[45] Rana K, Kent JL, Page A. Housing inequalities and health outcomes among migrant and refugee populations in high-income countries: a mixed-methods systematic review. BMC Public Health. 2025;25(1):1098. 10.1186/s12889-025-22186-5 PMC11929249 40121396

[46] Eveleigh ER, Nixon L, O’Donoghue M, Singh P, McDonald R, Ucci M, Sheringham J. Interconnected factors influencing family health and wellbeing in overcrowded homes and points for intervention – A qualitative study in London. Wellbeing, Space Soc. 2025;8:100250.

[47] Holding E, Foster A, Lumley E, Gilbertson J, Roxby S, Portman D, et al. Exploring the Impact of a Housing Support Service on Hospital Discharge: A Mixed-Methods Process Evaluation in Two UK Hospital Trusts. Health Soc Care Community. 2023;2023(1):4027277.

[48] Shelter. Making improvements to your rented home. 2023. Accessed online at: https://england.shelter.org.uk/housing_advice/repairs/making_improvements_to_your_rented_home

