# Supplementary material for: Associations between housing tenure and unscheduled hospital use in people of working age with multiple physical and mental long-term conditions: a retrospective cohort study using linked data from London
Source: BMC Health Serv Res. 2026 Jul 20;26:1417. doi: 10.1186/s12913-026-15071-3 (PMC13673713; doi:10.1186/s12913-026-15071-3)
Supplement: Supplementary file 1 [file 12913_2026_15071_MOESM2_ESM.docx]

Mahadevan et al. Associations between housing tenure and unscheduled hospital use in people of working age with multiple physical and mental long-term conditions: a retrospective cohort study using linked data from London

**Supplementary Data File 2**

**Supplementary Table 1. Physical and Mental Health Conditions**

Patients with a primary diagnosis of one of the following physical health conditions and mental health conditions included in the cohort adapted from the Academy of Medical Sciences (28). Mental health conditions consist of common mental illnesses listed in NICE guidance (29). Physical multimorbidity was identified using a simple unweighted count of physical health conditions (0, 1, 2, 3, or ≥ 4). We included the presence of any common mental health conditions as a binary variable (0 or 1).

| **Physical Health conditions** | **Mental Health conditions** |
| --- | --- |
| *Hypertension* | *Anxiety Disorder* |
| *Painful condition* | *Depression* |
| *Asthma (currently treated)* |  |
| *Coronary heart disease* |  |
| *Cancer (solid organ, haematological and metastatic)* |  |
| *Treated dyspepsia* |  |
| *Diabetes* |  |
| *Thyroid disorders* |  |
| *Rheumatoid arthritis, other inflammatory* |  |
| *Polyarthropathies & systematic connective tissue disorders* |  |
| *Chronic obstructive pulmonary disease* |  |
| *Irritable bowel syndrome* |  |
| *New diagnosis of cancer within last five years* |  |
| *Treated constipation* |  |
| *Stroke & transient ischaemic attack* |  |
| *Chronic kidney disease* |  |
| *Diverticular disease of intestine* |  |
| *Atrial fibrillation* |  |
| *Peripheral vascular disease* |  |
| *Heart failure* |  |
| *Prostate disorders* |  |
| *Glaucoma* |  |
| *Epilepsy (currently treated)* |  |
| *Inflammatory bowel disease* |  |
| *Migraine* |  |
| *Blindness & low vision* |  |
| *Chronic sinusitis* |  |
| *Bronchiectasis* |  |
| *Parkinson’s disease* |  |
| *Viral hepatitis* |  |
| *Chronic liver disease* |  |

**Supplementary Table 2. Description of all variables used in the analysis**

|  | **Type of Variable** | **Categories** |
| --- | --- | --- |
| **Exposure variable** |  |  |
| Housing Tenure | Categorical | 3 categories: Owner Occupied, Privately Rented, Social Housing |
| **Outcome Variables** |  |  |
| Unplanned admissions (NEL) | Integer | Binary |
| Length of Stay for NEL admissions | Numeric | Continuous |
| **Covariates** |  |  |
| Age | Numeric | Continuous |
| Sex | Binary | 2 categories : Male, Female |
| Ethnicity | Categorical | 6 categories : White, Asian, Black, Mixed, Other, Unknown |
| BMI | Ordered categorical | 6 levels: Underweight, Normal weight, Overweight, Obese, Morbidly Obese, Unknown |
| Smoking Status | Categorical | 4 categories : Ex-smoker, Non-smoker, Smoker, Unknown |
| Recorded Alcohol Consumption | Binary | Yes/no |
| Number of long-term conditions (see S2) | Character | 4 levels:1,2-3,4-5,>5 |
| Annual total number of prescriptions | Ordered categorical | 5 levels: 0-50,51-100,101-150,151-200,>200 |
| Year | Numeric | 2017,2018,2019 (based on financial years) |
| ***Household and area-level variables*** | | |
| Household occupancy | Ordered categorical | 4 levels: 1,2-4,5-9,10-12 |
| Index of Multiple Deprivation | Ordered categorical | 6 levels in deciles with least deprived combined: 1,2,3,4,5,6-10 |

**Supplementary Table 3. Sensitivity Analysis: Two-part model using complete case analysis and removal of admissions with length of stay less than 1 day to assess non-elective admission risk and stay duration**

|  | ***Unadjusted*** | | ***Adjusted^b^*** | |
| --- | --- | --- | --- | --- |
| Housing Tenure | PART 1-Logit OR (95 CI%) | PART 2 -GLM^a^  Coefficient (95 CI%) | PART 1-Logit OR (95 CI%) | PART 2 -GLM  Coefficient (95 CI%) |
| Owner- occupied (ref) |  | | | |
| Social Housing | 1.93(1.65-2.25)*** | 0.57(-0.42-1.56) | 1.80(1.53-2.13)*** | 0.08(-1.03-1.19) |
| Privately rented | 1.19(1.00-1.43)**^.^** | 0.83(-0.32-1.98) | 1.20(1.00-1.44)* | 0.59(-0.60-1.77) |

^a^GLM coefficients refers to the average length of stay

^b^Model adjusted for: Age, Gender, Ethnicity, Index of Multiple Deprivation (IMD),Year, Body Mass Index (BMI), smoking status, recorded alcohol intake, Number of physical-mental long-term conditions, Household occupancy

***p-value ≤0.0001	**p-value ≤0.001	*p-value ≤0.01**^.^**

**Supplementary Table 4. Results of Monte Carlo errors for all pooled coefficients in the Multiple Imputation**

| **Parameter** | **MCE** | **Pooled SE** | **MCE % of SE** |
| --- | --- | --- | --- |
| Ethnic group: Other | 0.0009 | 0.1641 | 0.5% |
| Ethnic group: Mixed | 0.0004 | 0.1226 | 0.3% |
| Ethnic group: Asian | 0.0001 | 0.0652 | 0.2% |
| Ethnic group: Black | 0.0001 | 0.0596 | 0.2% |
| Intercept | 0.0000 | 0.1437 | 0.0% |
| Age group: 25–34 | 0.0000 | 0.1111 | 0.0% |
| Age group: 35–44 | 0.0000 | 0.1067 | 0.0% |
| Age group: 45–54 | 0.0000 | 0.1075 | 0.0% |
| Age group: 55–64 | 0.0000 | 0.1086 | 0.0% |
| Tenure: Private Rented | 0.0000 | 0.0516 | 0.0% |
| Tenure: Social | 0.0000 | 0.0470 | 0.0% |
| IMD: 2nd decile | 0.0000 | 0.0813 | 0.0% |
| IMD: 3rd decile | 0.0000 | 0.0869 | 0.0% |
| IMD: 4th decile | 0.0000 | 0.1243 | 0.0% |
| IMD: 5th decile | 0.0000 | 0.1210 | 0.0% |
| IMD: 6th decile | 0.0000 | 0.1815 | 0.0% |
| IMD: 7th–10th decile | 0.0000 | 0.3522 | 0.0% |
| Gender: Female | 0.0000 | 0.0393 | 0.0% |
| Alcohol problem | 0.0000 | 0.0910 | 0.0% |
| Smoking: Ex-smoker | 0.0000 | 0.0547 | 0.0% |
| Smoking: Smoker | 0.0000 | 0.0492 | 0.0% |
| BMI: Morbidly Obese | 0.0000 | 0.0591 | 0.0% |
| BMI: Obese | 0.0000 | 0.0562 | 0.0% |
| BMI: Overweight | 0.0000 | 0.0560 | 0.0% |
| BMI: Underweight | 0.0000 | 0.1106 | 0.0% |
| PMLTC count: >5 | 0.0000 | 0.0932 | 0.0% |
| PMLTC count: 2–3 | 0.0000 | 0.0549 | 0.0% |
| PMLTC count: 4–5 | 0.0000 | 0.0641 | 0.0% |
| Occupancy group: 10–12 | 0.0000 | 0.2587 | 0.0% |
| Occupancy group: 2–4 | 0.0000 | 0.0547 | 0.0% |
| Occupancy group: 5–9 | 0.0000 | 0.0631 | 0.0% |
| Year: 2018 | 0.0000 | 0.0416 | 0.0% |
| Year: 2019 | 0.0000 | 0.0554 | 0.0% |
| Ethnic group: Other | 0.0084 | 1.4977 | 0.6% |
| Ethnic group: Mixed | 0.0040 | 1.1210 | 0.4% |
| Ethnic group: Asian | 0.0010 | 0.6066 | 0.2% |
| Ethnic group: Black | 0.0007 | 0.5421 | 0.1% |
| Intercept | 0.0003 | 1.3260 | 0.0% |
| Age group: 25–34 | 0.0001 | 1.0153 | 0.0% |
| Age group: 35–44 | 0.0002 | 0.9643 | 0.0% |
| Age group: 45–54 | 0.0002 | 0.9683 | 0.0% |
| Age group: 55–64 | 0.0002 | 0.9821 | 0.0% |
| Tenure: Private Rented | 0.0001 | 0.4781 | 0.0% |
| Tenure: Social | 0.0001 | 0.4333 | 0.0% |
| IMD: 2nd decile | 0.0001 | 0.7439 | 0.0% |
| IMD: 3rd decile | 0.0001 | 0.7939 | 0.0% |
| IMD: 4th decile | 0.0002 | 1.1453 | 0.0% |
| IMD: 5th decile | 0.0002 | 1.1120 | 0.0% |
| IMD: 6th decile | 0.0003 | 1.6858 | 0.0% |
| IMD: 7th–10th decile | 0.0004 | 3.2340 | 0.0% |
| Gender: Female | 0.0000 | 0.3637 | 0.0% |
| Alcohol problem | 0.0001 | 0.7992 | 0.0% |
| Smoking: Ex-smoker | 0.0002 | 0.5018 | 0.0% |
| Smoking: Smoker | 0.0002 | 0.4521 | 0.0% |
| BMI: Morbidly Obese | 0.0001 | 0.5467 | 0.0% |
| BMI: Obese | 0.0001 | 0.5171 | 0.0% |
| BMI: Overweight | 0.0001 | 0.5175 | 0.0% |
| BMI: Underweight | 0.0002 | 1.0047 | 0.0% |
| PMLTC count: >5 | 0.0001 | 0.8247 | 0.0% |
| PMLTC count: 2–3 | 0.0001 | 0.5037 | 0.0% |
| PMLTC count: 4–5 | 0.0001 | 0.5824 | 0.0% |
| Occupancy group: 10–12 | 0.0002 | 2.4288 | 0.0% |
| Occupancy group: 2–4 | 0.0000 | 0.5054 | 0.0% |
| Occupancy group: 5–9 | 0.0001 | 0.5839 | 0.0% |
| Year: 2018 | 0.0000 | 0.3847 | 0.0% |
| Year: 2019 | 0.0000 | 0.5106 | 0.0% |

*MCE = Monte Carlo error; SE = Standard error; IMD = Index of Multiple Deprivation; PMLTC = physical and mental long-term conditions; BMI = body mass index*
